# Staff retention in echocardiography: a formal exploration of workforce drivers

**DOI:** 10.1186/s44156-026-00137-z

**Published:** 2026-11-02

**Authors:** Carla Croft, Charlotte Barry, Macarena Fernandez Diaz, Wafa Sehailia, Delfin Encarnacion, Sanjeev Bhattacharyya, Guy Lloyd

**Affiliations:** 1 Barts Health NHS Trust, Trust Executive Offices, Ground Floor, Pathology and Pharmacy Building, The Royal London Hospital, 80 Newark Street, London, E1 2ES UK; 2 University College London Hospitals NHS Foundation Trust, London, UK; 3 East London NHS Foundation Trust, London, UK; 4 North London Cardiac Operational Delivery Network, London, UK; 5 Queen Mary University of London, London, UK; 6 Cleveland Clinic London, London, UK

**Keywords:** Echocardiography, Echocardiographers, NHS workforce, Staff retention, Intention to leave, Job satisfaction, Workplace wellbeing, Burnout, Psychological support

## Abstract

**Background:**

Retention of diagnostic imaging staff is a growing concern within the NHS, with echocardiography services facing particular pressure due to high demand, workforce shortages, and post-pandemic backlogs. While factors influencing NHS staff retention are well documented, limited research has focused specifically on echocardiography staff, a highly skilled and specialised workforce.

**Aim:**

This study aimed to explore intention to leave among NHS echocardiography staff and to identify factors that encourage retention, alongside perceived barriers to staying within the role or organisation.

**Methods:**

As a part of business as usual, Trust staff support psychologists offered to meet with echocardiography colleagues for a general support session and to advertise the services they offer. The psychological support aspect of the team session has an evidence base in the Trust that was used to form the session content. All echocardiography staff were invited to attend a facilitated two-hour group or individual sessions combining survey completion, structured discussion, and psychological support. Quantitative data on job satisfaction and intention to leave were collected alongside qualitative discussion data, which were anonymised and analysed thematically. This mixed-methods service evaluation was conducted within a large acute NHS Trust.

**Results:**

Forty-five staff participated, including Band 7 echocardiographers (*n* = 37) and Band 8 team leaders (*n* = 8). Retention intent differed by role: 88% of Band 8 leaders reported low intention to leave, compared with 39% of Band 7 staff indicating potential intention to leave within two years. Key retention factors included supportive management, positive team relationships, individualised flexibility, professional autonomy, and individualised plans around work–life balance. Barriers to retention centred on workload pressure, burnout (including musculoskeletal injury risk), limited career progression, and financial concerns. Younger staff were more likely to report thoughts of leaving. Participants highlighted meaningful relationships with patients and colleagues as protective against disengagement, alongside concerns regarding role narrowing and future AI integration. Impressions during the psychological support aspect involved the individualised understanding of flexibility, work-life balance and therefore a variety of potentially helpful solutions. The connection between colleagues generated in the group seemed of benefit.

**Conclusions:**

Retention among echocardiography staff is influenced by a complex interaction of organisational, professional, and wellbeing factors rather than pay alone. Multifaceted, individualised, targeted retention strategies focusing on workload management, career development, psychological support, and clear communication around service evolution are essential to sustaining this critical workforce.

## Introduction

NHS services focused on diagnosing and monitoring heart disease are at risk. Specifically, retention of diagnostic imaging and support staff is a critical issue as these staff face mounting work pressures. According to the British Society of Echocardiography, the echocardiography workforce is experiencing a “perfect storm” of high demand, backlog pressures and workforce attrition, with many staff reporting overwork, not being able to meet demands, low morale, fatigue and burnout [4].

The broader NHS picture is similar. There are widespread concerns about staff intention to leave their roles, and factors that influence the decision to stay or go. For example, a national survey found that nearly half (48%) of all NHS healthcare workers had considered leaving their role or taking early retirement [6].

Research indicates drivers for intention to leave the NHS are multifactorial. Recently, dissatisfaction with pay was identified as a key retention factor, with two-thirds of staff focusing in on improved pay as a strong incentive [7].

A prospective cohort study of NHS staff during the COVID-19 pandemic found that those who did not feel supported by colleagues or managers, who experienced mental-health difficulties, and who were younger were significantly more likely to report thinking about leaving or actively seeking employment outside their profession [9].

A meta-analysis of data from 79 workforce projects including over 46,330 health professional participants between 2019 and 2023 found that work satisfaction goes far beyond individual factors, however. This data showed general work environment plays a major role in decisions to leave, with the main themes being organisational support, recognition, workload, staffing levels and how work is managed [5].

Within the NHS acute sector specifically, an analysis by the Institute for Fiscal Studies found that leave rates fluctuate and vary considerably between staff groups, and that there is limited clear causal evidence on what drives retention. The study highlighted that individual, regional and organisational factors are all significant [3]. Furthermore, it is well-established that exposure to workplace violence (verbal or physical) and other traumatic experiences like racial discrimination within NHS hospital staff significantly increase intention to quit [6, 10].

Focusing on specific professional groups can help clarify the picture somewhat. For example, research on UK nurses historically revealed that relationships with leaders and perceived organisational support were strong predictors for intention to stay, mediated by the nurse’s reported individual emotional commitment to their organisation [8].

### Rationale and aim

Despite this growing body of research focusing in on the NHS workforce crisis and staff retention, fewer studies focus specifically on staff working in echocardiography though ongoing challenges in retention and satisfaction exist. Given the technical skill, high demand for throughput in echocardiographic services, and the post-pandemic and still emerging backlog, the retention challenges for this cohort require investigation. The British Society of Echocardiography warns that shortages in recruitment, training and retention threaten patient-safety and the sustainability of these vital services [4].

This study aims to explore the intention of echocardiography staff within the NHS to leave their roles, alongside the factors that encourage them to stay and the barriers to staying. By focusing on this specialist workforce, the research aims to deepen understanding of retention dynamics in a highly pressured diagnostic environment, and to identify actionable insights to inform workforce planning, service delivery and staff support strategies within echocardiography. This study also aims to contribute information about themes that can be generalised to other specialisms within the NHS and beyond.

## Methods

### Study design

This evaluation explored factors influencing staff retention and wellbeing among echocardiographers working within a large acute NHS Trust. The project combined psychoeducational and reflective practice components with a structured opportunity for participants to discuss job satisfaction and career intentions. The aim was twofold: (1) to offer facilitated group psychological support that has an evidence base in the Trust for benefiting staff wellbeing, and (2) to gain insight into factors contributing to engagement, satisfaction, and retention in echocardiography roles.

### Participants and setting

All echocardiography staff (N = approximately 60 whole time equivalents) employed across the Trust were invited to participate. The Trust comprises five varied hospital sites (that includes two general hospitals, a large teaching hospital and one specialist cardiovascular hospital). Many echocardiographers routinely work in more than one location. Participation was voluntary, and all attendees were released from normal clinical duties for a two-hour session.

Participants included echocardiographers at NHS Agenda for Change Band 7 (Echocardiographers) and Band 8 (Team Leaders). Four group sessions were conducted with Echocardiographers, each involving approximately ten participants. Two staff members opted to attend an individual session rather than a group session. One separate group session was held for Band 8 Team Leaders. In total, 45 echocardiography colleagues took part.

### Procedure

Each session was facilitated by a Practitioner Psychologist from the Trust’s Psychological Support Service, with the majority of sessions co-facilitated by a Trainee Clinical Psychologist. Sessions lasted approximately two hours and followed a semi-structured format. The first hour focused on data collection via survey and discussion regarding job satisfaction, engagement, and intention to remain within the Trust or the echocardiography profession more broadly. Participants individually completed a brief questionnaire covering these topics and then took part in an open group discussion. Key themes arising from the discussion were documented in real time by the facilitator or co-facilitator on a flipchart and later collated by these facilitators for thematic analysis.

Before each session, participants provided informed consent for anonymous use of discussion notes and questionnaire data in future reports and publications. They were advised that recording or reporting would cease after the first hour, at which point the support component of the session would begin.

### Context of the psychological support aspect

The second hour was dedicated to psychological support and wellbeing enhancement. This component began with a grounding exercise and included psychoeducation on stress management in the NHS context, reflective practice, and the sharing of individual coping strategies. Participants were reminded that this part of the session was confidential and not included in the data analysis. Information was provided for participants wishing to access further psychological support or contribute additional feedback privately at a later stage. The content of the session was based on five years of support work the psychological support team have conducted across the Trust with a wide range of NHS colleagues. It was presented to these echocardiography colleagues as a taster session and an introduction to psychological services available to them.

No measures were taken of the impact of the taster session. However, five years of service audit shows the psychological support team have worked with 2,809 different individuals for one-to-one support and have offered 29,801 points of contact in teams across the Trust sites. 83% of service users who have given feedback stated support from the service helped them cope better in their role (*n* = 574) and 97% of service users receiving psychological support giving feedback would recommend it to a colleague (*n* = 884). This work has also been externally evaluated and peer-reviewed, showing contact with the psychological support team significantly reduced symptoms of burnout and general stress, and improved mental wellbeing and team connection compared with colleagues who had no contact and when using before and after measures over four months. Additionally, absenteeism and intention to leave improvement was found for those who experienced positive wellbeing change [2].

### Data management and analysis

Notes taken during the open discussions were anonymised at the point of collection. Questionnaire responses and recorded themes were collated and stored on a secure Trust server accessible only to the evaluation team. Descriptive summaries were produced, and qualitative data were analysed thematically to identify recurrent patterns related to job satisfaction, stressors, and retention drivers among echocardiography staff.

### Governance, ethics and consent

This project was conducted as a service improvement and evaluation project. The work was designed to evaluate and inform local service delivery and did not constitute research as defined by NHS Health Research Authority guidance. The project did not involve patients or members of the public and included only NHS staff volunteer participants. No identifiable personal data was collected at any stage. Informed verbal consent for use of anonymised data was obtained from all volunteer participants prior to data collection. Written consent was not sought in order to preserve anonymity, as collecting written consent would have required the retention of identifiable information, which was intentionally avoided in the study design. Information governance support was required to approve the storage of the anonymised feedback data that was collected. GDPR does not apply as no personal data or data relating to an identifiable person was retained.

## Results

### Team leaders

The group of eight Band 8 leaders surveyed represents a diverse and experienced workforce, aged between 37 and 65, with significant tenure both within the Trust (4–19 years) and in their professions (15–29 years). See Table 1. The group includes a roughly even gender split and a mix of self-described ethnic backgrounds, highlighting the breadth of experience and perspectives among this senior leadership group.

Overall, retention among Band 8 leaders appears very strong, with 88% indicating they are *unlikely* to leave. Key factors contributing to this stability include a setting that can provide a good work–life balance, flexibility, supportive management, good team dynamics, fair pay, and opportunities for professional growth. Leaders also express a clear sense of passion for their work and value feeling acknowledged in their roles. See Table 2.

However, several potential risks to retention were identified by the leadership group. These include burnout, stress, and dissatisfaction with certain aspects of the role, particularly where managerial responsibilities feel excessive or expectations unclear. Broader issues such as AI integration, immigration concerns, and increasing workload pressures without corresponding resource growth could also potentially contribute to frustration and disengagement. Maintaining support structures and clear communication around expectations and future developments emerge as crucial to sustaining satisfaction and retention in this group.

### Band 7 echocardiographers

This survey of 37 staff members revealed a workforce that is diverse, experienced, and primarily composed of individuals under 40 years old (Table 3). Despite evidence indicating a motivated and diverse workforce, retention is a concern, with 39% indicating potential intention to leave within two years. The findings emphasise the role of individualised supportive work environments, career development, and flexible conditions in order to sustain engagement and satisfaction. However, issues such as workload, burnout including physical risk, limited career progression, and financial pressures pose tangible risks to staff retention (Table 4).

### General impressions from the support session

As mentioned a strong evidence base exists for the psychological support and wellbeing aspects of the sessions and no formal data was collected in this part of the session [2]. However, three strong qualitative impressions were observed by facilitators:

1. the meaning of concepts like “flexible working” and “good work-life balance” was individual.

For example, colleagues gave examples of flexible working allowances in their roles that varied widely (i.e., descriptions of helpful rota patterns that differed between individuals).

2. receiving individualised, targeted specialist support for workplace difficulties that had arisen for participants had indeed helped.

A number of people mentioned benefits of accessing physiotherapy support both inside and outside of the Trust, including general massage treatment. Additionally, some mentioned an awareness of the psychological support on offer and already benefiting from it.

3. time for connection with colleagues supports restoration.

There was resounding feedback across the sessions that the opportunity to connect with their colleagues for the two hours was powerful, with the majority of participants reflecting that they rarely have time to be together, to keep connections strong, and that they know these links protect them. There was a general message that participants were grateful for being given the session time.

## Discussion

Sonographer recruitment and retention remains a challenge for the NHS, with the Society of Radiographers recently reporting to parliament a 12.6% vacancy rate in diagnostic services [11]. Within echocardiography specifically, many departments across the country depend on locum staff and the British Society for Echocardiographers warns that without well-informed and focused retention efforts, the workforce will remain unstable [1].

This study illuminates strong positive reasons why professionals wish to stay and adds to research that indicates intention to leave is multifactorial, rather than focused on individual features. There is an indication that pay grade has an impact but, like other studies have shown, feeling supported by colleagues, managers and the organisation is key. Workload presents a significant barrier, linked with many other themes such as physical and mental wellbeing and engagement and passion. Younger staff also seem to be more likely to think of leaving, which mirrors data from other sources.

A number of echocardiographers mentioned that as their workload and demands have increased, their jobs have become narrower and less varied. In this context they also have less ability to prioritise their colleagues and their patients’ experience. Many mentioned the nourishing and informative connection with patients and colleagues as the key to restoration and helping them to carry on. This is even more of a priority with the advent of AI.

### Study limitations

This research was conducted with a group of physiologists working for a single trust that spreads across two district general hospitals, one large teaching hospital and a quaternary cardiovascular hospital. Though this variety across the sites gives the data a wide scope, the service does run as a single department which is unique and may make generalisation more difficult. Additionally, the Trust is located in one area of London, a specific context that has unique features such as more options for employment. The authors acknowledge that broader population data would strengthen the ability to draw conclusions. At the time of data collection (Summer 2025), though this has been a time of strain and post pandemic catch-up, local informatics showed all echocardiography posts were filled and the service was operating without significant backlogs, which could have had an impact in a variety of ways on the reflections of the participants. The authors believe this has been achieved via multi-modal strategies that circle back to the feedback on retention reflected in this research. Namely it is believed this stability has been achieved through a sustained focus on internal progression, and proactive workforce management rather than reliance on short‑term recruitment solutions. This has included investing in staff development pathways (specifically Band 6–7 progression and providing specialist accreditation support), providing consistent clinical supervision and leadership presence, and offering individualised flexible working arrangements to support work–life balance where possible. Vacancies have been minimised through early engagement with staff at risk of leaving, prompt backfilling of posts within establishment, and supporting redeployment, internal moves and training opportunities to enable growth. A focus on creating a strong team culture and reduced reliance on agency staff has also helped.

### Implications for NHS services

These findings show that simply focussing on one area in an effort to improve retention is insufficient and unlikely to be successful for this staff group. Targeted interventions across a broad base, focusing on support for enhancing both physical and mental wellbeing, improved opportunity for professional growth, individualised flexibility that provides a good perceived work-life balance and reward structures will therefore be instrumental in maintaining workforce stability. Key areas need to be considered both up front and with periodic reinforcement as part of a clear recruitment and retention strategy.

Specifically improving the clarity and accessibility of pathways for promotion, career development and financial incentives would be impactful. General working conditions impact the workforce in many ways and should also be a focus. Specifically considering factors like excessive workload that reduce the quality of patient and staff experience, and therefore satisfaction for all, is vital. Musculoskeletal injury is potentially linked to the point of overwork and is mentioned as a key driver for longevity in the profession. Planning that offers individualised flexible work patterns and that takes into account risk of burnout, that also invests generally in personal work-life balance, is key. Furthermore, departments need to spell out a clear roadmap to AI integration to allay fears of job replacement or down-skilling.

**Table 1:** Individual personal characteristics of the Band 8 team leader group

Age	37–65	
Time at Trust	4–19y	
Time in Profession	15–29y	
Gender	Male	3
	Female	4
	Not stated	1
Ethnicity	English/British	3
	White Other	2
	Asian Other	2
	Asian Filipino	1

**Table 2:** Band 8 retention, satisfaction and challenges

88% *Unlikely* to Leave within two years (7 out of 8 either somewhat or extremely unlikely)
HELPERS FOR STAYING
• Work–life balance & flexibility
• Supportive managers & teams
• Fair pay & career development
• Passion & acknowledgment
CHALLENGES / BARRIERS TO RETENTION
• Lack of support / burnout / stress
• Dissatisfaction with role expectations
• Repetitive or managerial workload
• AI and immigration concerns
• Increased demands without resources

**Table 3:** Band 7 echocardiographer demographics (*n* = 37)

Age range	21–65 yrs
Age	21–30: 15
	31–40: 16
	41–50: 5
	51–65: 1
Time at Trust	<3 months–19 yrs
Time in profession	2–40 years
Gender:	6 Male
	22 Female
	9 Not Stated
Ethnicity	Not Stated=8
	Filipino *n*=7
	Indian *n*=6
	English/British=4
	Other Asian=4
	Other White=3
	Mixed (White & Asian)=2
	Chinese=1
	Iranian= 1
	Irish=1

**Table 4:** Band 7 retention, satisfaction and challenges

39% (14 of 36) answered *Likely*,* Somewhat or Extremely Likely* to Leave Within 2 Years
HELPERS FOR STAYING
• Supportive work environment
• Career development & progression
• Flexible, predictable working conditions
• Financial incentives
• Sense of purpose & passion
CHALLENGES / BARRIERS TO RETENTION
• Physical strain & Musculoskeletal issues
• Burnout, workload & pressure
• Lack of flexibility
• Limited career progression
• Inadequate pay & cost of living
• Travel & commute issues
• Workplace dynamics

## Data Availability

All data available as required.
